# Community resilience and Chagas disease in a rural region of Mexico

**DOI:** 10.1590/S1518-8787.2016050005911

**Published:** 2016-07-26

**Authors:** José Antonio Santana Rangel, Luz Arenas Monreal, Janine M Ramsey

**Affiliations:** I Escuela de Salud Pública de México. Instituto Nacional de Salud Pública. Morelos. México; II Centro de Investigaciones en Sistemas de Salud. Instituto Nacional de Salud Pública. Morelos. México; III Centro Regional de Investigación en Salud Pública. Instituto Nacional de Salud Pública. Morelos. México

**Keywords:** Chagas Disease, prevention & control, Resilience, Psychological, Social Adjustment, Community Participation, Qualitative Research

## Abstract

**OBJECTIVE:**

To explore the pillars of community resilience in a region where Chagas disease is endemic, with the aim of promoting participatory processes to deal with this condition from the resilience of the population.

**METHODS:**

Qualitative study using ethnographic record and six interviews of focus groups with young people, women and men. The research was carried out in a rural area of the state of Morelos, Mexico, between 2006 and 2007. We carried out educational sessions with the population in general, so that residents could identify the relationship between the vector *Triatoma pallidipennis*, the parasite (*Trypanosoma cruzi*), symptoms, and preventive actions for Chagas disease. The ethnographic record and groups were analyzed based on Taylor and Bogdan’s modification, and the focus was to understand the socio-cultural meanings that guide the speeches and activities of residents in relation to the pillars of community resilience.

**RESULTS:**

The population felt proud of belonging to that location and three pillars of community resilience were clearly identified: collective self-esteem, cultural identity, and social honesty. Having these pillars as bases, we promoted the participation of the population concerning Chagas disease, and a Community Action Group was formed with young people, adult men and women, and social leaders. This Group initiated actions of epidemiological and entomological surveillance in the community to deal with this problem.

**CONCLUSIONS:**

It is necessary to create more experiences that deepen the understanding of the pillars of community resilience, and how they contribute to enhance participation in health to deal with Chagas disease.

## RESUMEN

**OBJETIVO:**

Explorar los pilares de la resiliencia comunitaria en una región en la que la enfermedad de Chagas es endémica, con la finalidad de partir de la resiliencia de la población para impulsar procesos participativos para enfrentar este padecimiento.

**MÉTODOS:**

Estudio cualitativo que utilizó registro etnográfico y seis entrevistas de grupos focales con jóvenes, mujeres y hombres adultos. La investigación se efectúo en una localidad rural del Estado de Morelos, México, entre 2006 y 2007. Se efectuaron sesiones educativas con la población en general, para que los habitantes identificaran la relación entre el vector *Triatoma pallidipennis*, el parásito (*Trypanosoma cruzi*), la sintomatología y acciones preventivas para la enfermedad de Chagas. El registro etnográfico y los grupos fueron analizados con base en una modificación de Taylor y Bogdan, y el foco fue comprender los significados socioculturales que guían los discursos y actividades de los pobladores en relación a los pilares de la resiliencia comunitaria.

**RESULTADOS:**

La población se sentía orgullosa de pertenecer a esa localidad y se identificaron con claridad tres pilares de la resiliencia comunitaria: autoestima colectiva, identidad cultural y honestidad social. Tomando como base estos pilares, se impulsó la participación de la población en torno a la enfermedad de Chagas, y se formó un Grupo de Acción Comunitaria con jóvenes, hombres y mujeres adultos, y líderes sociales. Este Grupo inició acciones de vigilancia epidemiológica y entomológica en la comunidad para hacer frente a esta problemática.

**CONCLUSIONES:**

Es necesario generar más experiencias que profundicen en la comprensión de los pilares de resiliencia comunitaria, y en la manera en que estos contribuyen a potenciar la participación en salud para enfrentar la enfermedad de Chagas.

## INTRODUCTION

The concept of resilience was adopted in the social sciences to refer to individuals or populations that, despite living in adverse conditions, are able to gracefully overcome different contexts^a^. The term is defined as the ability to face difficulties in a socially acceptable way^23^. Studies on resilience have focused, above all, at the individual level in specific situations^5,16,25^. Another focus has been the community context. Authors such as Melillo and Suárez Ojeda point out that the pillars of community resilience are: collective self-esteem, cultural identity, social or State honesty, social mood, and spirituality^13^. For the context of Latin America, and specifically Mexico, these pillars are reflected in everyday situations due to the strength of cultural aspects. Nevertheless, the pillar of social honesty represented by the State and institutions is problematic and absent in many cases. Norris et al. reinforce that social capital, economic development, community skills, information, and effective communication contribute decisively to community resilience when populations face natural disasters^17^. Few studies identified the characteristics of community resilience, and how they help the population face and recover from adversity^7,17^.

Chagas disease is one of the major neglected tropical diseases of the American continent^10,26^. It affects approximately between 16 and 18 million people, and it is estimated that 100 million live in endemic areas, in 21 countries^18^. It is the parasitosis of greatest backwardness in Latin America^6^, particularly in Mexico, where it has remained invisible thanks to the historic rejection to diagnose and address it among 1.0%-1.5% of the infected population^12^. Despite the social and cultural determinants that intervene to prevent the incidence of disease, research on the transmission and control of the vector has focused on the biology and parasite-vector binomial, leaving aside the contribution of human behavior and living conditions, which generate risk of exposure and vulnerability of transmission^22^. In Mexico, the politic and sanitary rejection of the disease has left a void of information for populations and the health sector, which is essential to prevent transmission. The parasite is zoonotic, which means that it is transmitted between approximately over 50.0% of small and medium-sized wild mammals in the country. When the vector requires alternative feeding, it uses and can transmit the parasite between human and domesticated animals. The ignorance of the existence of the disease and how it is transmitted, coupled with the omnipresence in over 75.0% of the Mexican territory of wild or domestic reservoirs infected with the parasite, and 28 species of the vector in all regions, indicates we are behind in the task to promote the prevention and care of between 1 and 2 million infected and diseased people^9,19^.

The objective of this study was to explore the pillars of community resilience in a region where Chagas disease is endemic, with the aim of promoting participatory processes to deal with this condition from the resilience of the population.

## METHODS

A qualitative design was employed, whose information gathering techniques included ethnographic record and focus groups. This research was conducted in a rural population of 2,200 inhabitants in the state of Morelos, Mexico, from September 2006 to August 2007, at the request of State Health Services, which had already identified Chagas disease as a problem in that locality. The research team was formed by master’s students of Public Health and by professors of the *Instituto Nacional de Salud Pública* (INSP).

The team kept contact with the population for 18 months, and lived in the community during the last six months of this study. This allowed the formation of a link with the daily life of the population, managing to establish ties of trust and mutual respect. At the same time that we collected the qualitative data, we carried out educational sessions with the population in general, so that residents could identify the relationship between the vector *Triatoma pallidipennis*, the parasite (*Trypanosoma cruzi*), symptoms, and preventive actions for Chagas disease.

### Participants

#### Focus groups

Six focus groups were formed with young people, women and men, two by population group. The two youth groups were formed with *telesecundaria* students, composed of nine and eight students. There were eleven girls and six boys, aged from 12 to 16 years, all single. The two groups of women were formed by beneficiaries of the *Oportunidades* Program, one by eight and the other by 10 participants, aged between 19 and 70 years. Of them, 18 were married, 15 had children, and two were single.

The first group of men was formed by six members aged between 55 and 76 years, all heads of their families; four were farm workers, one a carrier, and the other a constructor. The second group was organized with six members of the construction commission. Five were married and had children, whereas the sixth was single and had no children. Three were farm workers, one was a government employee, one was a blacksmith, and the latter was unemployed.

The study was approved by the Ethics and Research Committee of the *Instituto Nacional de Salud Pública*. All participants signed an informed consent form.

## Analysis

For the analysis of the ethnographic record and the data collected in the focus groups, it was crucial to understand the socio-cultural meanings that guide the speeches and activities of residents regarding self-esteem, collective identity, social honesty, spirituality, social capital, economic development, and community responsibilities. The analysis was performed based on a modification of the proposal of Taylor and Bogdan^21^, developed in two stages.

The stage of discovery was developed with the following activities: (i) repeatedly read the data to become intimately familiar with the contents; (ii) keep track of themes, insights, interpretations, and ideas evident to the superficial observation, i.e., open; (iii) delve into the themes, insights, interpretations, and ideas, in search of emerging issues; (iv) develop typologies, concepts, and theoretical propositions; and (v) highlight the main themes of the data. In the coding stage, the activities included: (1) develop codes; (2) read the material and apply the codes; (3) separate the data belonging to different categories of coding; (4) refine the analysis and coding, and separate the data, comparing different fragments related to each theme, concept, and proposition. To monitor the assumptions of the research team, a critical self-reflexion was performed to distinguish the perspectives and assumptions of the team itself^8^.

The central category that emerged from the analysis was community resilience. This study only presents data related to collective self-esteem, cultural identity, and social honesty.

## RESULTS

The results are presented in two sections: the first shows the systematization of the ethnographic record and the second the results from the focus groups.

### Proud to belong to Tetecalita

According to the ethnographic observation, agriculture was the reason why the locality was founded. Ownership of the land underwent great setbacks that have forged the history of the community identity. The residents pointed out that since the Viceroyalty, a real certificate of land ownership was given to the community. However, during the independent and revolution times, the property was uncertain until the official creation of the *Ejido* of Tetecalita.

“... basically, agriculture was promoted here since the foundation; above the community has done a lot of fighting to retain the lands and defend those rights (regarding the land)” (Mr. C).

Ownership of the land is distributed based on the *ejido* and on communal property, which is the reserve of land for common use or for descendants of the native inhabitants (Agrarian Reform). The community has a strong attachment to the agricultural activity, i.e., the land is linked to the identity of the people and of the locality:

“... I was born here and have the joy of having here my goods (my land)... I am used to the place; I have always fought very hard... so that we can better value and do better things in the village” (Mr. I).

Even today, the main economic activity is agriculture. The company Flora Plant is the main source of employment for most of the inhabitants; it employs over 300 of the 2,200 inhabitants of the village. For most of them, the village of Tetecalita presents excellent material conditions for the development of a satisfying life:

“Because of its natural conditions for living, its hills, its climate; first the land, it is very provident and the water used for drinking is not very saline” (Mr. V).

The previous statement denotes community self-esteem. The population highlights the main social resource of Tetecalita: the people, their skills and potentials:

“And its people, its people is really hardworking, that they are!” (Mrs. C).

“It is a specially dedicated to work people, the people live dedicated to work, with the desire to always improve their lives, their economy” (Mr. R).

In the village, an atmosphere of harmony was created, among natives and among those who came from other places:

“We have people here that had grandparents from different parts of the Republic; we have people who were born here but who emigrated due to their jobs, such as: the engineer specialist in sucrose Maurilio Cuellar; the doctor in sociology Raúl Rojas Soriano; another that was a coronel of the army, Rodolfo Soriano; and so there are many people who have left, but intelligent people also came to live here” (Mr. V).

In addition to the rooting to the village due to its means of subsistence and human resources, there are others factors that make the population proud to live in Tetecalita; one of them is its cultural tradition:

“... here everyone works together for the Feast of September 21. Some help with cooperations (money), and others with the cleaning. Women bring food to those who are working and then all help” (Mrs. L).

“There are many things; also the culture of Tetecalita is different from Chinconcuac or Xochitepec, of many places; traditions here are the feast of San Mateo, the *posadas* and the customs of the people that... If a person dies the neighbors bring bread, food, *atole*, whatever, to help the family who are suffering with the loss of their loved one” (Mr. R).

Other resources come from the honesty of the people and the union of the population:

“I chose to live here by calm, because I was not born here... I mean only one thing: previously, our village was all of palm cottages, many had small houses without door, they could leave their houses like this and nothing happened. Here people dedicate themselves to work and also to help one another and, well, here we are not strangers, we are neighbors and almost brothers” (Mr. R).

Another proof of the union of the population to solve their problems was identified during the ethnographic observation. At various times, we observed the *cuadrilla de faenas* [chores’ gang], a group of men organized by the assistantship to perform general maintenance actions for the community.

#### Focus groups

##### Collective self-esteem

The meaning of belonging to the village of Tetecalita reflects pride, regarding both the village and the people who inhabit it:

“I think, personally, that Tetecalita is a very special place and that it is not only a village, but it is everything for me: nation, country, because it is what I have at my hands’ reach, and I owe it. If you have to defend it, you have to defend it, but how? Working, trying to somehow serve those that formed this community. To me, that would be Tetecalita” (focus group men one: 48-71).

“Well, I, most of all, like this village a lot, I also like it because it is very quiet here, there are never thefts, well, at least at the time that I have been here it never happened to my house... I always leave everything like this... everything open and go out with confidence, and I come home to find everything in order; people, here are not crafty, they are calm, it is true” (focus group women: 126-139).

Part of the pride of the inhabitants of this community lies in the way in which people interact in everyday life:

“In some places, people are not united, do not support each other; then, this occur here, because some people feel sad or alone, and others approach them, make them feel better” (focus group young people one: 94-99).

##### Cultural identity

The interviewees referred to the significance of their people in the following manner: (1) a place where solidarity, participation and harmonious coexistence between people stands out, which leads to pride and satisfaction; (2) a place with culture, traditions, and identity; (3) the tranquility they have in the village. We found deep rootings to the village in the majority of the population groups.

“And here in the community, the good thing is that we know almost everyone, that is good because we know each and everyone of the people, and in a city it is very different, because at best they do not even open their doors to us...” (focus group women two women: 58-72).

“Also one may feel supported by all other people just saying I live in Tetecalita...” (focus group young people one: 71-76).

“Very nice, because we live in the same place and we get to know people little by little, and we coexist and so we have the opportunity to meet more people...” (focus group young people one: line 60-65).

In general, it is accepted that in situations of misfortune there is always collaboration to the affected. Other factors that act as favoring mechanisms to identity were the religious festivals celebrated in the village.

“As the party of the village stands out the most, as in other parts, in those days there is no other party” (focus group young people one: 432-436).

“... for example, a person dies and people participate a lot by bringing some small stocks, because this is the custom here when someone dies... such as coffee, chocolate, the bread, and the week that runs from prayers also carries an expense, cakes, chocolates, *tamales*, and then we unite, and many people get involved, because each person bring their bag of things to help in that way” (focus group women two: 2863-2879).

##### State or social honesty

We found that among the various characters that represent some authority in the village, the health center nurse was perceived as the most honest, for which she enjoys high prestige among the population. Regarding the other authority characters, the perception varies in the different population groups. The figure of the municipal assistant (local authority) is less prestigious.

“More than anything, yes, because she (the nurse) has already over 20 years of profession, and she knows well directly the majority of those who live here: patients, family members, all of them; she has always lived together with everyone for all of those 20 years working here and she knows everyone” (focus group men two: 391-401).

During the stay of the research team in the village, the election for municipal assistant took place, and votes were divided among five candidates. The difference between the first and the second places was one vote, and of 15 regarding the third place. This can be related to the absence of real or natural political leaders and to the generation of divisions in large groups within the community.

Starting from the identification of the pillars of resilience and the establishment of ties of trust and respect with the community, the research team promoted the organization around Chagas disease, and formed a group of community action, which was formed by four young people, six adult women, five adult men, the health center nurse, a primary school teacher, a professor at the *telesecundaria* and the municipal assistant. This group initiated actions of epidemiological and entomological surveillance concerning the Chagas disease in the community.

## DISCUSSION

This research identified the pillars of community resilience, and from them, promoted the strengthening of the participation of the population to deal with Chagas disease. This approach is new concerning vector-borne diseases. Cultural identity and community self-esteem, as defined by Melillo and Suárez Ojeda as pillars of resilience^13^, were taken as community resources to enhance the organization and to promote actions against Chagas disease. In this study, we found the pillar of social or State honesty to be weakened, which is a reflection of the current situation of Mexico, which has stablished itself in the last 15 years as one of the countries with high index of perception of corruption^4,24^. This leads to a weakening of the social fabric, and is reflected in community resilience. Although the population of this study considered their nurse honest and committed, this perception is built towards the person representing the nurse, and not the institution health or the State.

The pillars of community resilience proposed by Melillo and Suárez Ojeda^13^ have coincidence points with the set of attributes pointed out by Norris et al.^17^ These authors point out the following capabilities related to community resilience: economic development, social capital, information, communication, and community competences. Social capital includes the sense of belonging and participation, among other aspects^17^. The proposal by Norris et al.^17^ focuses mainly in the analysis of community resilience in situations of natural and social disasters, which is a difference regarding the proposal by Melillo and Suárez Ojeda, which focuses on identifying community resilience in daily life situations^13^. In this study, the presence and exposure to the vector *T. cruzi* is considered a risk, such as the environmental phenomena that cause disasters.

The results of this research indicate the importance of culture in community resilience, which is similar to that reported by different authors who have worked with young migrants in the United States and indigenous people in Australia and New Zealand^15^. The concept of community resilience is complex because it combines the meaning of community, to which converge aspects of physical, socio-cultural and economic environment, which interact with each other in a complex way. In this discussion it is recurring to hear that “the whole is more than the sum of its parts”, which means that a group of resilient people does not guarantee a resilient community. In addition to this, since reality is changing, resilience is a dynamic process^17^.

In contrast, several studies of resilience in the field of education highlight the urgent need to build communities resilient to strengthen resilience in children, adolescents, teachers, and schools^2,5^. These studies show that a resilient community with a context rich in participatory experiences that meet basic human needs, encourages the development of individual and community resilience.

Some studies on community resilience focuses on the view that people have of the world and in their socio-cultural, historical, and environmental backgrounds to promote the participation of the population^7,15^. Our study followed the same line, in which the cultural identity and the history of the community played a fundamental role.

To face Chagas disease in Latin America, the most common have been vertical programs with the application of pesticides by health teams^14,20^. This restricts the participation of a segment of the population who regularly collaborates more than actively participates. Abad et al.^1^ reported that, for the control of Chagas disease, the active participation of the population is fundamental, along with decision makers and social actors of decisions, so that control strategies to this problem can be planned^1^.

This study surveyed the socio-cultural, historical, and environmental context of the population, based on the pillars of community resilience, to enhance participation as an alternative to deal with Chagas disease. Therefore, we discouraged the use of pesticides, given that this activity sends a message of dependence on the State and encourages the passivity of the communities. By the generation of an educational process on the triatomine and Chagas disease, the population managed organizational and participatory processes.

Regarding diseases transmitted by vectors, in particular Chagas disease, to generate organizational processes arising from the strengths of the population is fundamental. In that sense, to investigate the pillars of community resilience is fundamental.

Some authors point out difficulties in relation to the concept of resilience used, both at the individual level and in the ecological and community level^3,11^. At the individual level, Luthar et al. point out that the difficulties focus on the ambiguities of the concept and the variability in measurement to determine resilience among people^11^. In the case of the concept of resilience in the ecological and community levels, Brown points out that there is little analytical incorporation of the management of power and of the social and politic dimensions^3^. Without denying the aforementioned aspects, the discussion today around resilience, both individual and community, has opened debates, reflections, and spaces to advance in the understanding of the way in which people and populations face adversity. In our study, we have taken as basis the characteristics of community resilience of authors such as Melillo and Suárez Ojeda^13^, Norris et al.^17^, and Mooney et al.^15^, who incorporated socio-cultural and political aspects to their analysis, although we recognize that the review of the management of power is still insufficient.

From public health, it is necessary to create more experiences that deepen the understanding of the pillars of community resilience, and how they contribute to enhance participation in health to deal with Chagas disease. To deal with Chagas disease it is essential to promote the active participation of the population, for which starting from community resilience, in spite of the difficulties that that may lead, can: strengthen organizational processes, increase participation in health, and deal comprehensively with this problem.

## Footnotes

a Kotliarenco MA, Cáceres I, Fontecilla M. Estado de arte en resiliencia. Washington (DC): OPS/OMS; 1997.
